# CMOS compatible high-Q photonic crystal nanocavity fabricated with photolithography on silicon photonic platform

**DOI:** 10.1038/srep11312

**Published:** 2015-06-18

**Authors:** Yuta Ooka, Tomohiro Tetsumoto, Akihiro Fushimi, Wataru Yoshiki, Takasumi Tanabe

**Affiliations:** 1 Department of Electronics and Electrical Engineering, Faculty of Science and Technology, Keio University, 3-14-1, Hiyoshi, Kohoku-ku, Yokohama 223-8522, Japan

## Abstract

Progress on the fabrication of ultrahigh-*Q* photonic-crystal nanocavities (PhC-NCs) has revealed the prospect for new applications including silicon Raman lasers that require a strong confinement of light. Among various PhC-NCs, the highest *Q* has been recorded with silicon. On the other hand, microcavity is one of the basic building blocks in silicon photonics. However, the fusion between PhC-NCs and silicon photonics has yet to be exploited, since PhC-NCs are usually fabricated with electron-beam lithography and require an air-bridge structure. Here we show that a 2D-PhC-NC fabricated with deep-UV photolithography on a silica-clad silicon-on-insulator (SOI) structure will exhibit a high-*Q* of 2.2 × 10^5^ with a mode-volume of ~1.7(*λ*/*n*)^3^. This is the highest *Q* demonstrated with photolithography. We also show that this device exhibits an efficient thermal diffusion and enables high-speed switching. The demonstration of the photolithographic fabrication of high-*Q* silica-clad PhC-NCs will open possibility for mass-manufacturing and boost the fusion between silicon photonics and CMOS devices.

Silicon photonics is a rapidly growing research field because it is expected to lead to a reduction in the power consumed by signal processing by allowing the integration of all-optical switches^1^,^2^, electro-optic modulators^3^, germanium detectors and CMOS electrical devices on a silicon chip^4^,^5^. The development of low-loss waveguides^6^,^7^ and high-*Q* microcavities^8^ was one of the technological breakthroughs that made it possible to build these photonic devices. However, the maximum *Q* and minimum *V* have been demonstrated so far with PhC nanocavities^9^, and these features are crucial if we want to realize Raman lasers^10^ and fabricate cavity quantum electro-dynamics devices^11^,^12^ that are the missing components in silicon photonics. The fusion of these two technologies (i.e. silicon photonics and PhC technology) will lead to a variety of uses including classical and quantum applications, but challenges remain to be overcome. First, a high-*Q* PhC nanocavity usually requires an air-bridge structure, which is both frangible and unstable. The creation of an air-bridge structure requires the removal of a sacrificial silica (SiO_2_) layer by isotropic etching, which makes it difficult to integrate PhC devices with other silicon photonics devices on the same chip, which are usually clad with SiO_2_. Secondly, a high-*Q* PhC nanocavity is usually fabricated by using electron-beam lithography, which is an accurate but time-consuming process. Although researchers have recently demonstrated high-*Q* PhC nanocavities with SiO_2_^13^ and spin-on-glass claddings^14^, whose demonstration is an important step towards fusion with silicon photonics, they used electron-beam lithography for the fabrication. Instead, we need to employ a stepper photolithography process if we hope to use our technologies for commercial applications. Photolithography is widely used for fabricating commercially available CMOS devices, and this CMOS process will make it much easier for us to integrate PhC devices with other silicon photonics and CMOS devices. PhC devices have been fabricated for waveguides using the CMOS process^15^,^16^,^17^ but there have been only a few studies with respect to nanocavities. Although a high-*Q* 1D-PhC nanocavity has been fabricated using KrF immersion lithography in the polysilicon layer of a bulk CMOS process^18^, loaded *Q* was ~10^4^ (unloaded *Q* is 6 × 10^4^) and we believe there is still plenty of room left to achieve a higher value. Furthermore, a 1D-PhC nanocavity usually has a slow operating speed when we use it for all-optical operation, because the carriers and heat cannot diffuse quickly outside the cavity unless complex structure such as *p*-*n* junction is made. Hence, a high-*Q* 2D-PhC nanocavity is advantageous if we can fabricate an ultrahigh-*Q* cavity on an SOI substrate.

## Results

### Design and fabrication of a silica clad PhC nanocavity

**Figure 1:**
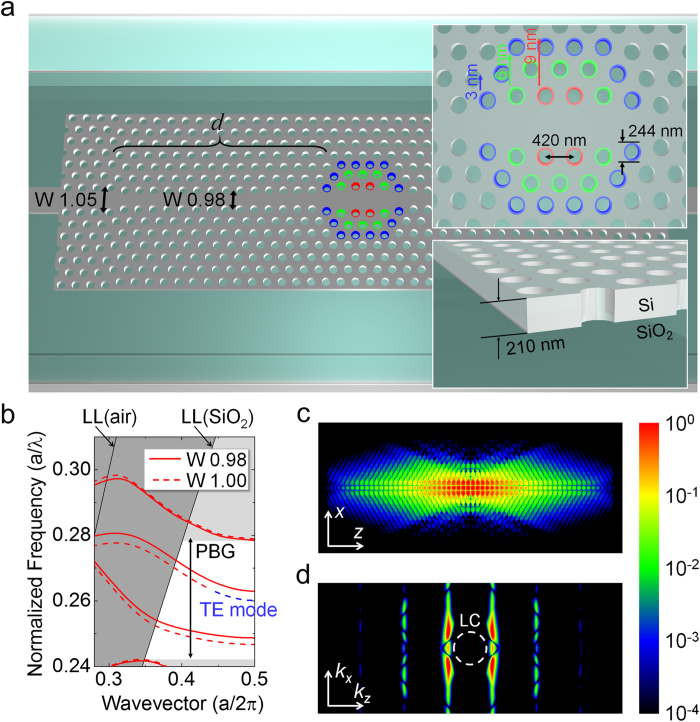
Design and calculation of a PhC microcavity. (**a**) Schematic illustration of a two-dimensional width-modulated line defect PhC nanocavity. Air holes only at the centre of the structure are slightly shifted toward the outside of the waveguide from their original position. The silicon slab is covered with SiO_2_. (**b**) Dispersion of a two-dimensional PhC waveguide (line defect) with SiO_2_ cladding. The light line (LL) for both the air and SiO_2_ claddings are shown in the graphs. The solid line is the dispersion of the W0.98 waveguides (without cavity hole shifts) that we use as barriers. The dashed line is when the waveguide width is 18 nm wider (with cavity hole shifts) than the barrier waveguide, which corresponds to the width of a W1 waveguide. (**c**) The calculated cavity mode profile (*H*_z_) of a width-modulated line defect with SiO_2_ cladding with three-dimensional FDTD. The *Q* is 7.1 × 10^6^ and the mode volume is ~2.4 (*λ*/*n*)^3^. (**d**) The *k*-space distribution of the cavity mode in (c). The white dashed line is the light cone (LC) of the SiO_2_ clad.

The highest *Q* yet reported in a PhC nanocavity was achieved with a mode-gap confined cavity, where the PhC waveguide was locally modulated. Figure 1a shows a schematic illustration of a width-modulated line defect cavity^19^, which is one of the designs capable of realizing mode-gap confinement. Figure 1b shows the band diagram for a PhC line defect. By increasing the width of the line defect we can shift the mode gap toward a lower frequency. As a result, a frequency component that is close to the mode gap (i.e. a wavevector component that is close to the Brillouin edge) will localize when we shift the hole only in a certain place of a PhC waveguide. Since the original PhC waveguide mode is lossless and the perturbation (i.e. the amount of hole position shift) is small, the vertical scattering is kept to a minimum, and hence we can obtain an ultrahigh *Q*. The problem as regards achieving a high *Q* in a 2D-PhC nanocavity, especially when the refractive index of the cladding material is high, is the difficulty of designing a cavity mode that has a small wavevector component in the light cone^20^. On the other hand, the wavevector of the localized mode of a width-modulated line defect is well below the light line even for SiO_2_ cladding as shown in Fig. 1b, and this allows us to realize a small vertical radiation loss. Therefore, with this design we can expect to obtain a very high *Q* even with SiO_2_ cladding.

With this in mind, we first performed a finite-difference time-domain (FDTD) calculation for a 2D-PhC nanocavity with SiO_2_ cladding. The calculated optical mode is shown in Fig. 1c, where we obtained a *Q* of ~7.1 × 10^6^ and a mode volume of ~2.4 (*λ*/*n*)^3^, when the hole shifts were 2, 4, and 6 nm (slab thickness 204 nm, lattice constant 420 nm, air-hole diameter 216 nm, refractive index of silicon 3.47, and refractive index of SiO_2_ 1.44). Figure 1d is the spatially Fourier transformed *k*-space distribution of Fig. 1c. Small component exist in the light cone (LC) of the SiO_2_, which tells us that the radiation loss to the SiO_2_ slab is low. When we calculated the *Q* of a cavity with the parameters that we used in our experiment (slab thickness 210 nm, lattice constant 420 nm, air-hole diameter 246 nm, shift = 3, 6, and 9 nm), we obtained a value of 8.1 × 10^5^ with a mode volume of ~1.7(*λ*/*n*)^3^. As expected, it shows that an ultrahigh *Q* is possible with an SiO_2_-clad 2D-PhC when we use a width-modulated line defect cavity.

**Figure 2:**
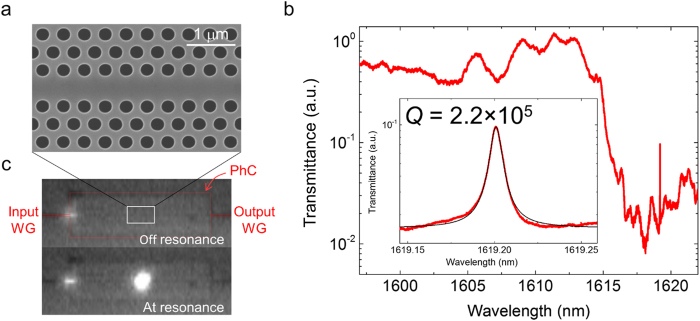
Observation of photoluminescence and transmittance spectra. (**a**) An SEM image view of the device from the top after the SiO_2_-cladding is removed. (**b**) The transmission spectrum of the nanocavity. A resonance is found at 1619.20 nm. The *Q* value is 2.2 × 10^5^. (**c**) Device images acquired from the top of the sample using an infrared camera. When the input wavelength of the laser light is 1619 nm (below the mode gap) there is no light scattering from the cavity region. When the input light is at 1619.20 nm (at the resonance of the cavity mode), we observed light scattering from the cavity. The silicon waveguides are indicated by the red squares.

Next, we sent the sample for fabrication with a standard CMOS process line. It is an open silicon photonics foundry service. It employs a photolithography based standard silicon photonic process, which allowed the silicon PhC to be integrated with other devices, such as a spot-size converter (SSC)^21^,^22^. The coupling loss between the fibre and the silicon waveguide of the fabricated device was 0.8 dB. Figure 2a shows a scanning electron microscope (SEM) image of the fabricated device. It has a slab thickness of 210 nm, lattice constant of 420 nm, and air-hole diameter of 246 nm. We observed that the PhC structure was fabricated with little error; the deviation of hole diameter was 1.6 nm.

### Transmittance spectrum of a high-*Q* nanocavity

Figure 2c shows the transmittance spectrum. There is a sharp edge in the transmittance spectrum at a wavelength of 1615 nm, which is due to the mode-gap barrier of a W0.98 PhC waveguide (98% of the original width). The cavity resonance was at 1619.20 nm. The transmittance was kept low to obtain a high loaded *Q*, but it can be controlled by changing the length of the W0.98 barrier PhC waveguides. The inset in Fig. 2c shows the transmittance of the resonance peak. From the measured result, we obtain a *Q*-factor of 2.2 × 10^5^. This is the highest *Q* reported for a PhC fabricated with a photolithographic process. To confirm that the light was trapped in the cavity, we recorded an infrared camera image from the top of the slab with the input laser off and at resonance as shown in Fig. 2b. It shows the clear confinement of the light in the cavity region. As has been demonstrated, a PhC nanocavity with a *Q* of 10^5^ will allow us to realize a variety of applications such as all-silicon detectors^23^ with a sensitivity of over 0.02 A/W and a sub-microwatt micrometre scale silicon Raman lasers^24^. The important feature of our structure is that these devices are integrated with other SiO_2_-clad silicon photonics devices.

### Demonstration of nonlinear switching

We describe all-optical nonlinear switching experiments that reveal the advantage of this device over existing PhC nanocavity devices. Demonstration of all-optical switching has been made with EB lithographic fabricated air-bride 2D PhC nanocavities^25^. A high-*Q* cavity has been obtained with a one-dimensional nanobeam cavity^13^, which is also an attractive platform for nonlinear applications. However, we expect our device to have two advantages over previous devices. First, we can improve the thermal property. Our structure can dissipate heat three-dimensionally through SiO_2_ cladding and through a 2D silicon slab. An air-bridge structure cannot dissipate heat in the vertical direction and a nanobeam cavity can dissipate it only one-dimensionally. Since the thermal conductivity of silicon (149 W/(m·K)) is much higher than that of SiO_2_ (1.05 W/(m·K)), the heat diffusion take place dominantly in silicon structures. However, the SiO_2_ cladding also contribute to a better thermal property since the thermal conductivity of air is very low (0.026 W/(m·K)). As a result, the thermo-optic (TO) effect is kept to a minimum in our device. Secondly, the operation is faster than that of other devices when we perform all-optical nonlinear switching based on carriers and heat due to the same reason. We performed two experiments to investigate these phenomena.

**Figure 3:**
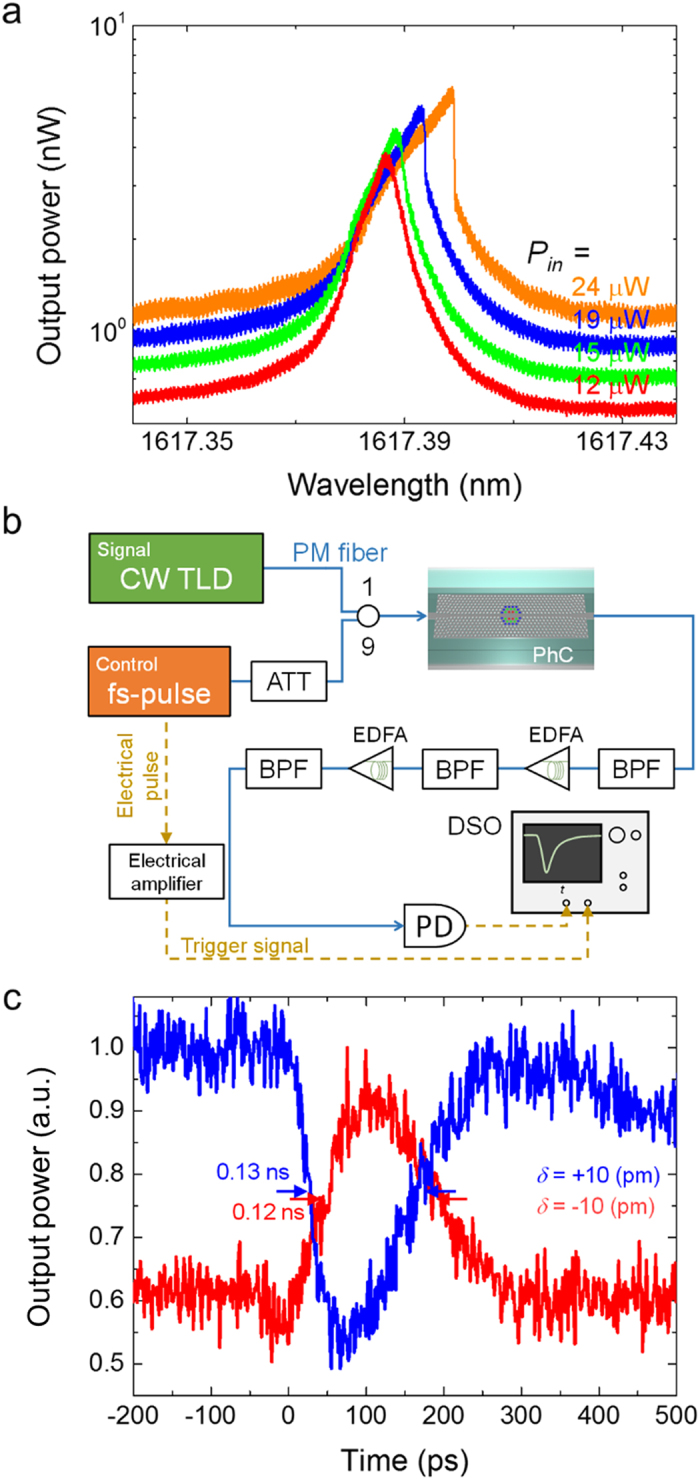
Nonlinear measurement. (**a**) Transmission spectra of the width-modulated cavity (*Q* = 2.1 × 10^5^) at different CW input powers *P*_in_ (shown in the graph). The centre wavelength shift is caused by the thermo optic effect, where the bistable threshold is at 19 μW. (**b**) Schematic illustration of the switching experiment. ATT: attenuator, DSO: digital sampling oscilloscope (20-GHz bandwidth), PD: photodiode (40-GHz bandwidth). BPF: band-pass filter. (**c**) All-optical switching experiment. Transmittance waveform of the signal light when the device is pumped with a picosecond pulse laser that is above the mode gap of the barrier line defects. The modulation is due to the carrier plasma dispersion effect caused by the excitation of the two-photon absorption carriers. The carrier lifetime is 0.12 ns.

Figure 3a shows the output spectrum from the nanocavity which has *Q* of 2.1 × 10^5^ at different input powers. The laser is swept from a short to a long wavelength, and we observe an asymmetric transmittance spectrum resulting from the TO bistability. The threshold power is 19 μW (intra-cavity power), which indicates that a 2D-PhC structure suffers much less from the TO effect than an 1D nanobeam cavity. Indeed the threshold value for an 1D nanobeam cavity was 1 μW when the *Q* is almost the same^13^. In addition, we measured the TO effect of an air-bridge sample to investigate the thermal diffusion through the SiO_2_ clad. When the SiO_2_ clad is removed, the threshold power decreased at 4.5 μW (intra cavity power) for a cavity with a *Q* of 2.3 × 10^5^. It indicates that the surrounding SiO_2_ clad also contribute to a smaller TO effect. The small TO effect is usually preferred in photonic devices.

Next, we investigated the speed of the device. We performed all-optical switching based on the generation of carriers by two-photon absorption. A fibre mode-locked femtosecond laser was injected into the device as a control. By measuring the transmittance of the continuous wave signal light, we can demonstrate all-optical switching and obtain the response time of the device. Figure 3b shows the setup and 3c is the measured waveform of the signal light, where we obtain an effective carrier lifetime of 0.12 ns. This value is much faster than that of a microring resonator^26^, where it is difficult for carriers to diffuse outside the device. Although surface recombination will alter the effective carrier lifetime, previous study shows that the effect is limited and the diffusion is the primal contribution to the fast speed^27^.

## Discussion

**Figure 4:**
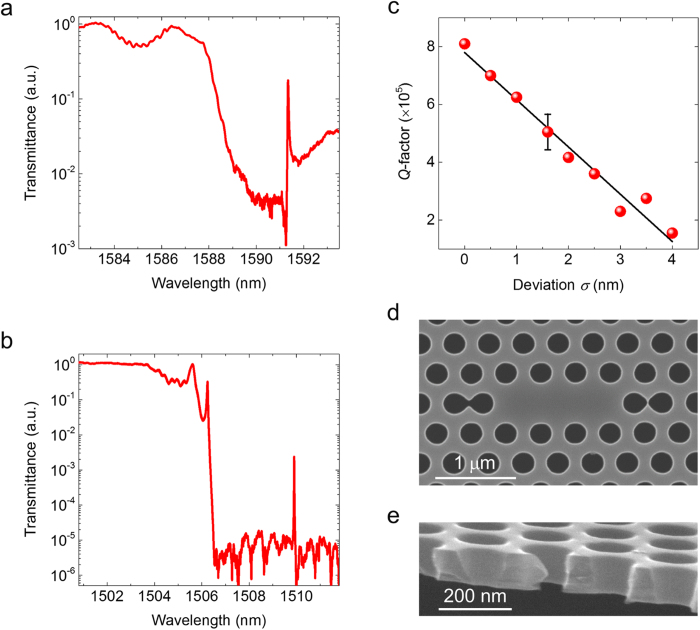
The effects of SiO_2_ cladding and photolithography. (**a**) Transmission spectrum of a width-modulated line defect cavity with SiO_2_ cladding. The barrier length is *d* = 9. (**b**) The same as **a** when the SiO_2_ cladding is removed with hydrogen fluoride and an air-bridge structure is formed. (**c**) Relationship between *Q*-factor and hole diameter deviation. *Q* is calculated by using 3D FDTD. Randomness is added to the air hole position of the PhC cavity with deviation *σ*. The red dots are the calculated result and the solid curve is the fitted line. *σ* is 1.6 nm for the fabricated device, so the calculation was performed 14 times for the point at which *σ* = 1.6 nm. (**d**) A SEM image of an L3 cavity that was fabricated on the same chip. The two end holes at the cavity ends are shifted 63 nm towards the outside to exhibit a high *Q*. However, these holes are seriously affected by the local modulation of an optical proximity effect. (**e**) An SEM image of the cross-section of the 2D PhC slab after SiO_2_ clad is removed by wet etching.

We investigate the reason for the difference between the experimental and theoretical *Q* values. Our device is different from those in other studies in two respects; it is covered with SiO_2_ and it is fabricated by photolithography. To determine which influence dominates the loss, we first removed the SiO_2_ cladding and studied the *Q* value. When the *Q* value of a clad cavity with *d* = 12 (number of air-holes between the cavity end and in/out waveguides) was 1.8 × 10^5^, we confirmed that the value improved to 2.4 × 10^5^ after the SiO_2_ cladding was removed and an air-bridge structure formed. Another trial was undertaken with a cavity where *d* = 9 and that exhibited a *Q* of 1.7 × 10^5^. The value was initially 4.6 × 10^4^ before the SiO_2_ cladding was removed. Figure 4a and b show the experimental results. The mode gap and the cavity resonance both shifted to a shorter wavelength because of the smaller effective refractive index of the slab. Note that the *Q* value in Fig. 4a is low because of the strong coupling with the waveguides; indeed we obtained an intrinsic *Q* value of ~9.7 × 10^4^ from the transmittance of the resonant mode. The coupling becomes small when we remove the SiO_2_ because of the stronger light confinement in an air-bridge slab. Indeed we observe a sharper mode gap in Fig. 4b. This is consistent with our FDTD calculation where we obtained a mode volume for a cavity with an SiO_2_-clad device [~1.7(*λ*/*n*)^3^] that was about 20% larger than with an air-bridge one [~1.4(*λ*/*n*)^3^]. But the important fact we obtained from this experiment is that the *Q* value of the air-bridge structure is limited to a few hundred thousand, which does not differ greatly from the highest *Q* obtained with an SiO_2_-clad cavity. Therefore, we conclude that the low experimental *Q* (compared with the theoretical value) has a different cause. When we look closely at the transmittance spectrum in Fig. 4b, we can see small peaks in the stop band of the transmittance spectrum at a wavelength of 1506 nm, which originate from the Anderson localization of light^28^,^29^,^30^. This indicates that some fabrication error is inherent in this device.

We therefore investigated the effect of the fabrication error. If the fluctuation at the waveguide is very large, we should observe multiple peaks even for an SiO_2_-clad device. However, neither Fig. 2b nor Fig. 4a contain such spectrum shapes. This already provides a clue suggesting that the structural fluctuation at the PhC waveguide is sufficiently small for us to use it as a platform for a mode-gap confined cavity. A comparison of the behaviour at the stop band in Fig. 4a and b indicates that the presence of SiO_2_ cladding makes the PhC nanocavity more insensitive to the structural fluctuation with some cost to the mode volume. To study the fabrication error in more detail, we carefully analysed the SEM picture in Fig. 2a. By measuring the radius of the holes, we obtained a fluctuation deviation *σ* of 1.6 nm, when the average diameter was 240 nm. To study the fabrication error tolerance of our width-modulated line defect cavity, we performed an FDTD calculation taking the randomness of the hole diameters into account. The result is shown in Fig. 4c. As expected, *Q* decreases when the fluctuation is larger. The *Q* value is 4.3 × 10^5^ when *σ* = 1.6 nm, and this value agrees with the value we obtained experimentally. This value of *σ* is a few times larger than the value obtained with EB lithography where a deviation of 0.36 ~ 0.58 nm has been reported^31^,^32^. We also investigated *Q* for an optimised structure. As discussed earlier the *Q* value is ~7.1 × 10^6^ for an optimised cavity when no fluctuation is present. When we added the same fluctuation of *σ* = 1.6 nm, we obtained a *Q* ranging from 6.0 to 8.4 × 10^5^.

Here we discuss on the accuracy of the resonant wavelength of the cavity. By measuring the transmission spectra of different samples that is on the same chip, we obtained a resonant wavelength fluctuation of 0.16 nm (20 GHz). Since this value is not far from the devices fabricated with EB lithography^2^, we are optimistic to be able to demonstrate wavelength dependent applications such as Raman lasers and cavity QED experiments in future.

Since we use photolithography, it is important for us to know that state-of-the-art fabrication technology based on photolithography meets the requirement to fabricate an ultrahigh-*Q* PhC nanocavity when we select a good cavity design.

One of the keys to obtaining an ultrahigh *Q* is to choose a design that is suited to the CMOS process. We were able to obtain a high *Q* with a width-modulated line defect cavity because only a slight perturbation of the hole position is needed to form a cavity. The structure is basically a uniform PhC W1 waveguide. As a result the structure does not exhibit a strong local proximity effect. Although the optical proximity correction is a powerful technique for fabricating small structures, it is still important to select a design that is less affected by the optical proximity effect. When the hole position and diameters are modulated larger, the proximity effect varies greatly at the cavity, which makes fabrication very difficult. Indeed, Fig. 4d shows an SEM image of an L3 PhC nanocavity, whose design should exhibit an ultrahigh *Q*. However, the fabricated structure shows that the two air holes at the edge of the cavity are connected with other air holes, and we were not able to observe resonance with this structure. We were able to obtain a high *Q* because we selected a width-modulated line defect cavity, which requires only a slight perturbation of the hole positions to form a cavity.

Finally we should add a few comment on the cross-section of the air-holes. One of the issues with foundry fabrication is that the etching process is not necessarily optimized for PhC air-holes. The cross-section at the edge of our PhC device is shown in Fig. 4e, where we observe tapered structure for the air-holes. It appears surprising to have a *Q* of >10^5^ with this structure. However, the 3D-FDTD study has revealed that the effect of the taper is limited. When the diameters on the top and the bottom of the silicon slab are 4-nm different, we obtained a high-*Q* of 7 × 10^5^ for a silica cladded device. Others report a *Q* of 6 × 10^6^ with an similar structure (air-bridged) when the diameter of top and the bottom differ for 9%^33^.

In summary, we have shown that a high *Q* value of 2.2 × 10^5^ can be obtained with a PhC nanocavity device on an SOI structure fabricated with a CMOS process when we use a width-modulated line defect nanocavity. This is the highest value for a PhC fabricated using photolithography. The fusion of PhC nanocavity technologies with Si photonics will advance significantly only when we develop a technology that can fabricate these devices with exactly the same process and structure (i.e. CMOS process and SiO_2_ cladding structure). Therefore, this study constitutes an important step toward the realization of the full integration of these devices on a silicon chip. In addition, we showed that an SiO_2_-clad PhC nanocavity has a good thermal property because the heat can diffuse three-dimensionally. This also contributes to a faster operating speed when we use these devices for nonlinear control. Our study shows that an even higher *Q* value ranging from 6 to 8.4 × 10^5^ may be possible by optimising the structure, and this would present new possibilities for silicon photonics technology.

## Methods

### Fabrication

We used an open silicon foundry service by IME (Singapore; Institute of Microelectronics), where the device was fabricated using KrF stepper lithography at a wavelength of 248 nm. The PhC is fabricated with a high resolution mask, which has a resolution of 180 nm. An 8-inch silicon-on-insulator wafer (SOITECH) with 210 nm thick silicon layer and a box layer of 2 μm was used, and it was over-clad with SiO_2_ at a thickness of about 2 μm once the silicon layer had been processed.

As mentioned in the main text, Fig. 1a is a schematic illustration of the fabricated PhC nanocavity. The hole diameter and the lattice constant of the PhC lattice were 246 and 420 nm, respectively. The input and output PhC waveguides were 105% of the original width (W1.05). The barrier waveguides had a 98% width (W0.98) to shift the mode gap towards a higher frequency. The barrier waveguide, *d*, was 15 periods long, which isolated the cavity well from the input/output waveguides (i.e. undercoupled condition). The holes at the cavities were shifted 3, 6, and 9 nm.

### Transmission spectrum measurement

A tuneable laser diode (Santec TSL-510, linewidth of 200 kHz) is used to measure the transmittance spectrum of our PhC device. A lensed fibre with a focal length of 0.2 mm is used to couple the light in a silicon nanowire via a spot size converter, and then the light reaches the PhC. The structure is symmetric, so the output is re-collected with a lens and coupled in a polarization maintaining fibre on the other side. Light is detected with a power meter (Agilent 81634B), while we scan the wavelength of the input.

### Measurement of all-optical switching

We used a fibre mode-locked femtosecond laser with a pulse width of 1.0 ps, an energy of 52 pJ, and a wavelength of 1561.3 nm and injected it into the device as a control. Since this wavelength is shorter than the mode-gap of the W0.98 line defects, it can propagate through the structure, exciting TPA carriers. At the same time, we injected the continuous wave signal light, which is at the resonance of the cavity or off resonant (*δ* = ±10 pm). We can demonstrate all-optical switching and obtain the response time of the device, measuring the transmittance of the signal light in time domain.

### Removal of SiO_2_ cladding

The SiO_2_ cladding was removed with hydrofluoric acid for the SEM observation and the experiment of TO effect in air-bridge PhC. The under cladding was removed by the distance (over 1 μm) enough not to affect PhC. The time of etching (11 min) was determined from the concentration of hydrofluoric acid (20%) and the temperature (23 °C).

## Additional Information

**How to cite this article**: Ooka, Y. *et al*. CMOS compatible high-*Q* photonic crystal nanocavity fabricated with photolithography on silicon photonic platform. *Sci. Rep.* **5**, 11312; doi: 10.1038/srep11312 (2015).
